# Identifying Key MicroRNAs Targeted by Narenmandula in a Rodent Nephropathy Model

**DOI:** 10.1155/2020/9196379

**Published:** 2020-11-24

**Authors:** Xiulan Wang, Chun Chang, Wenjie Jin, Arun Arun, Sudunabuqi Sudunabuqi, Aodaofu Aodaofu, Xiaowei Liu, Fengjiao Wu, Hongmei Chen

**Affiliations:** ^1^Mongolia Medical Department of Inner Mongolia University for the Nationalities, Inner Mongolia Autonomous of China, Tongliao 028000, China; ^2^Affiliated Hospital of Inner Mongolia University for the Nationalities, Inner Mongolia Autonomous of China, Tongliao 028000, China

## Abstract

**Background:**

Untreated nephropathy can progress to renal failure. The traditional Mongolian remedy Narenmandula regulates the kidney “yang.” This study aimed to identify key microRNAs (miRNAs) targeted by Narenmandula in a rat model of nephropathy.

**Methods:**

Fifteen rats exhibiting normal renal function were randomized to three study arms. Nephropathy was induced in *n* = 10 rats using doxorubicin hydrochloride, followed by either Narenmandula treatment (treatment group) or no treatment (control group). In *n* = 5 rats, no doxorubicin was given and renal function remained unchanged (healthy group). Microarray analysis identified miRNAs which were differentially expressed (DE-miRNAs) between groups. Target genes of DE-miRNAs were predicted using miRWalk version 2.0, followed by enrichment analysis using DAVID, and construction of the miRNA coregulatory network using Cytoscape.

**Results:**

Nephropathy was successfully induced, with doxorubicin resulting in differential expression of 3645 miRNAs (1324 upregulated and 2321 downregulated). Narenmandula treatment induced differential expression of a total of 159 miRNAs (102 upregulated and 57 downregulated). Upregulated DE-miRNAs (e.g., miR-497-5p, miR-195-5p, miR-181a-5p, miR-181c-5p, and miR-30e-5p) and downregulated DE-miRNAs (e.g., miR-330-3p and miR-214-3p) regulated a high number of target genes. Moreover, the miRNA pairs (e.g., miR-195-5p—miR-497-5p, miR-181a-5p—miR-181c-5p, and miR-30e-5p—miR-30a-5p) coregulated a high number of genes. Enrichment analysis indicated functional synergy between miR-30e-5p—miR-30a-3p, miR-34a-5p—miR-30e-5p, miR-30e-5p—miR-195-3p, and miR-30a-3p—miR-195-3p pairs.

**Conclusion:**

Narenmandula may modulate doxorubicin-induced nephropathy via targeting miR-497-5p, miR-195-5p, miR-181a-5p, miR-181c-5p, miR-30e-5p, miR-330-3p, miR-214-3p, miR-34a-5p, miR-30a-3p, and miR-30a-5p.

## 1. Introduction

Chronic nephropathy is a progressive disorder, which when untreated can result in renal failure, including consequent uremia and death [1, 2]. The main risk factors for nephropathy are glomerular immunoglobulin A (IgA) deposition, analgesic consumption, xanthine oxidase deficiency, and cytotoxic chemotherapeutic drugs [3]. Globally, nephropathy impacts quality of life in millions of patients, and thousands of these require life-saving renal transplantation [4]. A more comprehensive understanding of the pathogenesis of nephropathy will contribute to development of more effective therapies and improved outcomes for nephropathy patients.

In recent years, nephropathy mechanisms have been partially elucidated by several studies. For example, in reflux nephropathy (RN), angiotensin-converting enzyme (ACE) overexpression induces tubulointerstitial damage via increased extracellular matrix (ECM) component production; ACE inhibition may thus suppress renal fibrosis in this context [5]. By suppressing activation of the v-akt murine thymoma viral oncogene homolog (AKT)/phosphatase and tensin homolog (PTEN) pathway, miR-21 inhibition restrains tubular cell and podocyte fibrogenesis in IgA nephropathy (IgAN) [6, 7]. Furthermore, miR-146a plays an anti-inflammatory role in the context of diabetic nephropathy (DN) [8], and miR-18a-5p modulates autophagy by regulating the ataxia telangiectasia mutated (*ATM*) gene, which may be prophylactic or therapeutic in the context of DN [9]. Moreover, miRNAs have exhibited utility as biomarkers and/or therapeutic targets in the context of renal disorders such as IgA nephropathy and DN [10–12]. Nevertheless, pathogenesis of nephropathies remains incompletely understood.

The traditional Mongolian remedy Narenmandula (also known as Sheng Yang Shi Yi Wei Wan) is a combination of pomegranate, cinnamon, cardamom, humble pie, yellow essence, safflower, wintergreen fruit, bamboo, asparagus, *Bletilla*, and *Tribulus terrestris* [13]. The major traditional uses of Narenmandula include regulating the kidney “yang”, promoting capillary refill and digestion, and relieving diarrhea and edema [14]. Although polysaccharides present in Narenmandula exhibit antioxidant activity [15], potential mechanisms of Narenmandula in opposing nephropathy have not been investigated.

In the present study, doxorubicin hydrochloride was used to induce nephropathy in rats, followed by treatment with Narenmandula. Microarray-based miRNA expression profiling of healthy, treatment, and control groups was followed by differential expression analysis, prediction of genes targeted by differentially expressed miRNAs (DE-miRNAs), enrichment analysis, and miRNA coregulatory network construction. Results regarding key miRNAs modulated by Narenmandula may contribute to identifying novel therapeutic targets in the context of nephropathy.

## 2. Materials and Methods

### 2.1. Model Establishment and Sample Acquisition

Study protocols were approved by the Ethical Committee of Affiliated Hospital of Inner Mongolia University for the Nationalities, Tongliao, Inner Mongolia Autonomous of China. Rats were obtained from Yisi Laboratory Animal Technology Co., Ltd. (Jilin, Changchun, China). After a 5-day acclimation period, 15 rats exhibiting normal total 24 h urine protein content were selected for randomization to a healthy group (receiving 6.5 mL/kg normal saline), treatment group (receiving 6.5 mg/kg doxorubicin hydrochloride, Shenzhen Arcandor's Pharmaceutical Co., Ltd., Guangdong, Shenzhen, China) followed after a 4-day period by 3.0 g Narenmandula (Tong Kang Pharmaceutical Co., Ltd., Hebei, Anguo, China) once per day for 21 consecutive days), and a control group (receiving doxorubicin followed by distilled water rather than Narenmandula). Each group contained *n* = 5 rats. Saline and doxorubicin were administered intravenously by injection into the tail caudal vein, while Narenmandula was administered intragastrically. After the final dose of Narenmandula or water, all rats were fasted for 12 h prior to anesthetization with pentobarbital. Animals were then euthanized via abdominal aortic phlebotomy.

### 2.2. Microarray Analysis and Data Preprocessing

An miRNA 4.0 Array (Affymetrix, Santa Clara, CA) was used to determine whole blood miRNA expression profiles of rats in each group. Expression data have been deposited in the Gene Expression Omnibus (GEO) database (accession number GSE123776). The Robust Multichip Average (RMA) algorithm in *R* package “affy” [16] was used in conjunction with CEL result files to conduct background correction (integrating probe and probeset signals) and normalization (minimizing the effect of between-sample biological variability).

### 2.3. Model Validation: Comparing Healthy and Treatment Groups

R package “limma” [17] was used to determine whether any miRNAs were differentially expressed between the healthy and treatment groups, using as screening criteria *|*log_2_ fold-change (FC)| > 0.58 and *p* value ≤0.05. Rodent nephropathy model success was defined as the presence of significant DE-miRNAs.

### 2.4. Comparing Treatment and Control Groups and Prediction of Target Genes of Identified DE-miRNAs

Differential expression analysis was again performed using *R* package “limma” and identical criteria. Genes targeted by DE-miRNAs were predicted using miRWalk version 2.0 [18]. Using combined predictive results from 12 databases, including miRWalk [19], miRanda [20], MicroT4 [21], miRDB [22], miRBridge [23], PICTAR2 [24], PITA [25], miRMap [26], miRNAMap [27], RNA22 [28], TargetScan [29], and RNAhybrid [30], DE-miRNA-target gene pairs were screened. Pairs predicted by ≥six databases were retained for downstream analysis.

### 2.5. Target Gene Enrichment Analysis

Using DAVID version 6.8 (with classification stringency set to medium) [31], Gene Ontology (GO) [32] and Kyoto Encyclopedia of Genes and Genomes (KEGG) [33] terms were predicted for target genes of miRNAs differentially expressed between treatment and control groups. The threshold for significance was set at *p* value <0.05.

### 2.6. Construction of the miRNA Coregulatory Network

Based on shared targets of miRNA pairs, an miRNA coregulatory network was constructed using Cytoscape version 3.4 [34]. For miRNA pairs coregulating the highest number of target genes, GO enrichment analysis was conducted. The threshold for significance was set at *p* value <0.05. Functional synergy was defined as significant enrichment of biological process (BP) terms between common targets of an miRNA pair [35].

## 3. Results

### 3.1. Model Validation: Comparing Healthy and Treatment Groups

**Figure 1:**
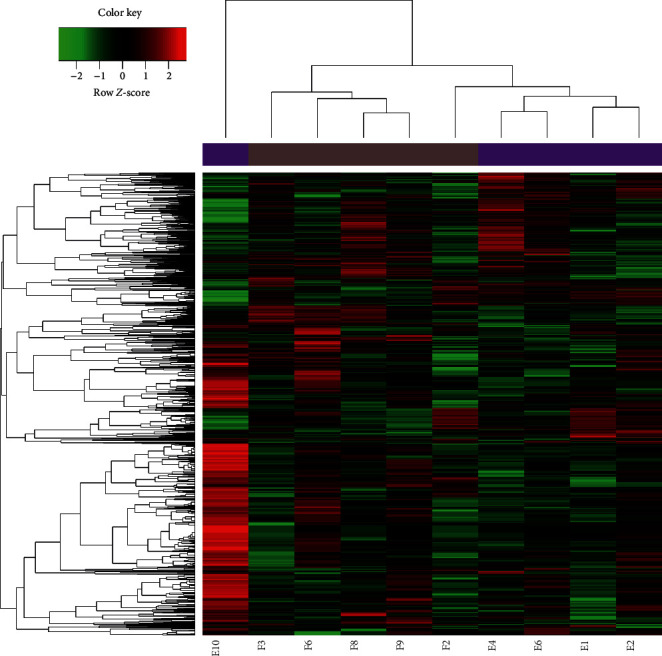
Clustered heat map of miRNAs differentially expressed between healthy (E) and treatment (F) groups.

When comparing healthy and treatment groups, 3645 miRNAs were significantly differentially expressed (1324 upregulated and 2321 downregulated in the treatment group; Figure 1), suggesting successful induction of nephropathy.

### 3.2. Comparing Treatment and Control Groups and Prediction of Target Genes of Identified DE-miRNAs

**Figure 2:**
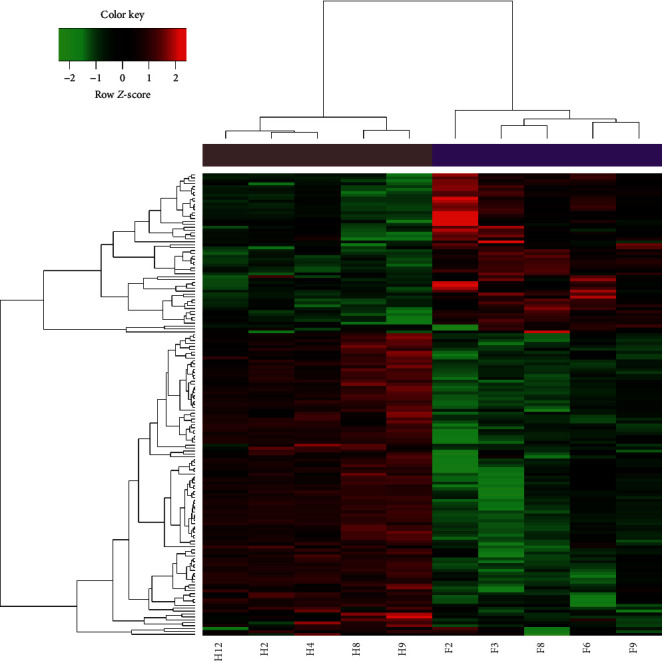
Clustered heat map of miRNAs differentially expressed between treatment (F) and control (H) groups.

When comparing treatment and control groups, 159 miRNAs were significantly differentially expressed (102 upregulated and 57 downregulated in the treatment group; Figure 2). Based on FC, the top ten most upregulated and downregulated DE-miRNAs are shown in Table 1.

Based on output from 12 databases, 17002 miRNA-gene pairs (involving 97 upregulated miRNAs) and 5582 miRNA-gene pairs (involving 53 downregulated miRNAs) were identified. Based on the number of target genes, the top ten upregulated DE-miRNAs (e.g., miR-497-5p, miR-195-5p, miR-181a-5p, miR-181c-5p, and miR-30e-5p) and downregulated DE-miRNAs (e.g., miR-330-3p and miR-214-3p) with the highest number of target genes are shown in Table 2.

### 3.3. Target Gene Enrichment Analysis

**Figure 3:**
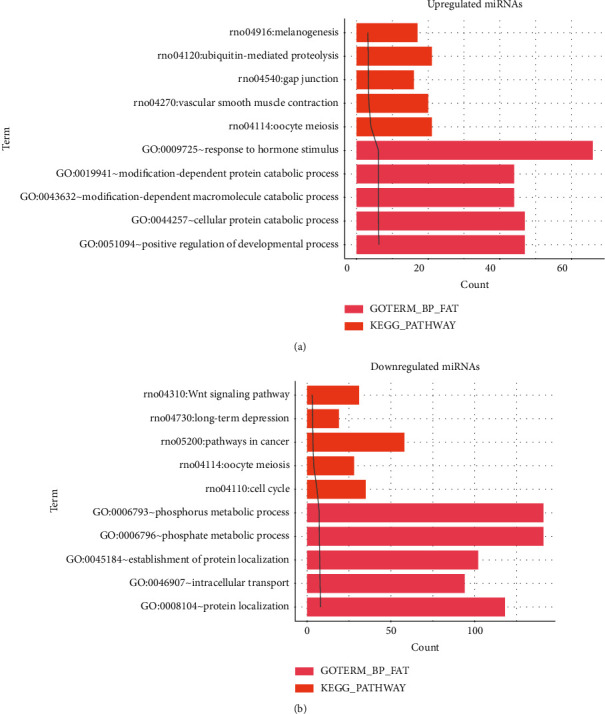
Top five enriched biological processes and KEGG pathways represented by target genes of upregulated miRNAs (a) and downregulated miRNAs (b). GO, Gene Ontology; KEGG, Kyoto Encyclopedia of Genes and Genomes; BP, biological process.

Biological process and KEGG biological pathway enrichment analysis of genes targeted by the top ten most significantly up- and downregulated DE-miRNAs (Figure 3) revealed that genes targeted by upregulated miRNAs are mainly involved in response to hormone stimulus (GO BP) and ubiquitin-mediated proteolysis (KEGG pathway) (Figure 3(a)) and that genes targeted by downregulated miRNAs are mainly involved in the phosphorus metabolic process (GO BP) and Wnt signaling pathway (KEGG pathway) (Figure 3(b)).

### 3.4. Construction of the miRNA Coregulatory Network

**Figure 4:**
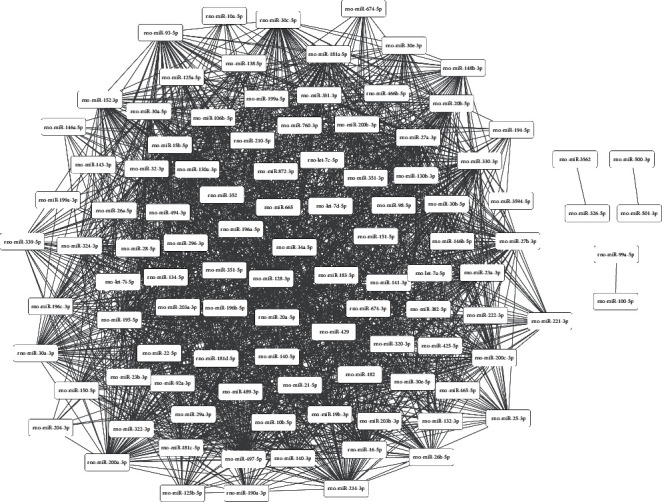
Constructed miRNA coregulatory network. Edge thickness indicates the number of common targets.

Shared genes targeted by miRNA pairs were identified (the top ten miRNA pairs coregulating the highest number of genes, such as miR-195-5p—miR-497-5p, miR-181a-5p—miR-181c-5p, and miR-30e-5p—miR-30a-5p, are shown in Table 3). Subsequently, an miRNA coregulatory network was constructed (Figure 4). To limit the size of the network, only miRNA coregulatory pairs with target gene count larger than ten were incorporated. Finally, GO BP enrichment of genes targeted by the top ten miRNA pairs coregulating the highest number of genes (including miR-30e-5p—miR-30a-3p, miR-34a-5p—miR-30e-5p, miR-30e-5p—miR-195-3p, and miR-30a-3p—miR-195-3p) (Table 4) demonstrated the presence of functional synergy between these miRNA pairs.

## 4. Discussion

The present study demonstrated that 159 miRNAs (102 upregulated and 57 downregulated) were significantly differentially expressed between treatment and control groups. Upregulated miRNAs (e.g. miR-497-5p, miR-195-5p, miR-181a-5p, miR-181c-5p, and miR-30e-5p) and downregulated miRNAs (e.g. miR-330-3p and miR-214-3p) targeted the largest number of genes. Furthermore, miRNA pairs (e.g., miR-195-5p—miR-497-5p, miR-181a-5p—miR-181c-5p, and miR-30e-5p—miR-30a-5p) coregulated the largest number of target genes. Finally, enrichment analysis suggested the existence of functional synergy between certain miRNA coregulatory pairs (e.g., miR-30e-5p—miR-30a-3p, miR-34a-5p—miR-30e-5p, miR-30e-5p—miR-195-3p, and miR-30a-3p—miR-195-3p).

Melatonin is known to alleviate endothelial-to-mesenchymal transition (EMT) of glomerular endothelial cells during DN by altering expression of miR-497 [36] (upregulated by Narenmandula in the present study). Overexpression of miR-497 can significantly repress proliferation, migration, and invasiveness of renal cancer cells and may serve as a prognostic factor and therapeutic target for this tumor type [37]. The present study demonstrates upregulation of miR-195 by Narenmandula. Decreased miR-195 expression prevents mesangial cell apoptosis, and miR-195 may also exert antiapoptotic effects during early DN [38]. The immunosuppressive drug cyclosporine A (CsA) is nephrotoxic, producing renal injury and fibrosis, but miR-181c (upregulated by Narenmandula) may help protect renal tissues against this [39]. However, miR-181a inhibition alleviates 5-fluorouracil- (5-FU-) associated nephrotoxicity and may represent a promising target for treatment of chemotherapy-induced nephrotoxicity [40]. Furthermore, miR-181a overexpression contributes to clear cell renal cell carcinoma (ccRCC) progression via modulating Krüppel-like factor 6 (KLF6) expression; targeting miR-181a may have therapeutic potential in this context [41, 42]. Also upregulated by Narenmandula in the present study, miR-30e exhibits low-level expression in DN, and overexpression can prevent progression of DN to renal fibrosis [43]. We speculate that miR-497-5p, miR-195-5p, miR-181a-5p, miR-181c-5p, and miR-30e-5p may be implicated in protective effects of Narenmandula in the context of nephropathy.

Regarding miRNAs demonstrated by the present study to be downregulated by Narenmandula, morin-induced miR-330 expression prevents abnormal fructose-induced renal insulin signal transduction and is a novel candidate for treating renal injury [44]. The twist/miR-214/E-cadherin axis in renal tubular epithelial cells (RTECs) plays roles in EMT, suggesting that anti-miR-214 therapy may slow development of renal fibrosis [45]. Cross-talk between PTEN and miR-214 attenuates glomerular hypertrophy, implying that miR-214 may be a promising target for treatment of DN [46]. Thus, miR-330-3p and miR-214-3p may also contribute to nephropathy-alleviating effects of Narenmandula.

Regarding miRNA pairs coregulating a large number of target genes, suppression of miR-30a-3p and miR-30c-2-3p promotes expression of hypoxia-inducible factor-2*α* (HIF-2*α*), which weakens HIF-1*α*-mediated ccRCC inhibition [47]. Furthermore, urinary miR-30a-5p (as well as miR-490 and miR-196a) levels are associated with focal segmental glomerulosclerosis (FSGS) activity, and miR-30a-5p predicts the response of active FSGS to steroid treatment [48]. Via the miR-34a-5p/sirtuin 1 (SIRT1)/HIF-1*α* pathway, long intergenic noncoding RNA (lincRNA) 1700020I14Rik suppresses expression of renal fibrosis biomarkers, inhibits mesangial cell proliferation under diabetic conditions, and is involved in progression of DN [49]. We thus speculate that Narenmandula impacts nephropathy via targeting of miR-34a-5p, miR-30a-3p, and miR-30a-5p.

In conclusion, key miRNAs, including miR-497-5p, miR-195-5p, miR-181a-5p, miR-181c-5p, miR-30e-5p, miR-330-3p, miR-214-3p, miR-34a-5p, miR-30a-3p, and miR-30a-5p, may be associated with protective effects of Narenmandula in nephropathy. However, Narenmandula-modulated expression of key DE-miRNAs in rat model renal tissues has not yet been orthogonally verified (e.g., using real-time PCR). Moreover, we did not measure urinary albumin or the albumin/creatinine ratio (ACR) in order to elucidate mechanisms by which key Narenmandula-modulated DE-miRNAs may oppose nephropathy. Additional research is therefore required to confirm our findings.

## Figures and Tables

**Table 1:** Top ten miRNAs differentially expressed between treatment and control groups.

miRNA	Log fold-change	*p* value	Type
rno-miR-34a-5p	3.507493097	7.83*E* − 07	Up
rno-miR-375-3p	2.8912011	1.88*E* − 04	Up
rno-miR-203b-3p	2.820823524	2.69*E* − 06	Up
rno-miR-132-3p	2.752535606	2.39*E* − 06	Up
rno-miR-196a-5p	2.702999341	3.79*E* − 04	Up
rno-miR-146b-5p	2.669461069	8.91*E* − 07	Up
rno-miR-203a-3p	2.582396551	1.19*E* − 06	Up
rno-miR-183-5p	2.487984128	6.41*E* − 04	Up
rno-miR-30e-3p	2.303984656	5.21*E* − 07	Up
rno-miR-155-5p	2.244312291	3.55*E* − 06	Up
rno-miR-32-3p	−2.110700334	3.19*E* − 05	Down
rno-miR-423-5p	−1.948686272	8.55*E* − 05	Down
rno-miR-466b-5p	−1.935924295	2.38*E* − 04	Down
rno-miR-214-3p	−1.894326482	5.88*E* − 07	Down
rno-miR-760-3p	−1.734602185	4.45*E* − 06	Down
rno-miR-138-5p	−1.722493294	1.31*E* − 03	Down
rno-miR-652-5p	−1.569177177	8.48*E* − 05	Down
rno-miR-92b-5p	−1.515952809	5.95*E* − 07	Down
rno-miR-500-3p	−1.453944308	3.84*E* − 05	Down
rno-miR-328a-5p	−1.439955519	1.31*E* − 07	Down

**Table 2:** Top ten differentially expressed miRNAs with the highest number of target genes.

Upregulated	Number	Downregulated	Number
rno-miR-497-5p	446	rno-miR-330-3p	408
rno-miR-15b-5p	384	rno-miR-214-3p	390
rno-miR-195-5p	357	rno-miR-93-5p	320
rno-miR-16-5p	354	rno-miR-320-3p	284
rno-miR-181a-5p	341	rno-miR-140-3p	252
rno-miR-181c-5p	335	rno-miR-32-3p	248
rno-miR-30e-5p	333	rno-miR-465-5p	216
rno-miR-128-3p	332	rno-miR-351-5p	204
rno-miR-30c-5p	331	rno-miR-324-3p	200
rno-miR-30b-5p	330	rno-miR-339-5p	185

**Table 3:** Top ten miRNA pairs coregulating the highest number of target genes.

miR1	miR2	Number
rno-miR-15b-5p	rno-miR-497-5p	355
rno-miR-195-5p	rno-miR-497-5p	334
rno-miR-497-5p	rno-miR-16-5p	332
rno-miR-195-5p	rno-miR-16-5p	326
rno-miR-15b-5p	rno-miR-195-5p	323
rno-miR-30b-5p	rno-miR-30c-5p	319
rno-miR-15b-5p	rno-miR-16-5p	317
rno-miR-181c-5p	rno-miR-181a-5p	313
rno-miR-30e-5p	rno-miR-30a-5p	309
rno-miR-30b-5p	rno-miR-30a-5p	300

**Table 4:** Enriched biological processes represented by gene targets of the top ten miRNA coregulatory pairs.

miR1	miR2	Cogene	Co-GO-BP
rno-miR-30a-3p	rno-miR-30e-5p	355	28
rno-miR-195-3p	rno-miR-32-3p	334	115
rno-miR-30e-5p	rno-miR-32-3p	332	120
rno-miR-30a-3p	rno-miR-465-5p	326	121
rno-miR-30e-5p	rno-miR-465-5p	323	94
rno-miR-32-3p	rno-miR-465-5p	319	67
rno-miR-34a-5p	rno-miR-30e-5p	317	99
rno-miR-30e-5p	rno-miR-195-3p	313	131
rno-miR-195-3p	rno-miR-465-5p	309	54
rno-miR-30a-3p	rno-miR-195-3p	300	55

GO, Gene Ontology; BP, biological process.

## Data Availability

Datasets generated by this study are available from the Gene Expression Omnibus (GEO) database (accession number GSE123776).

## Additional Points

(1) There were 159 dysregulated miRNAs between treatment and control groups. (2) Several miRNA coregulatory pairs exhibit functional synergy. (3) Narenmandula may modulate nephropathy via targeting key miRNAs.
